# Investigating the Toxicity, Uptake, Nanoparticle Formation and Genetic Response of Plants to Gold

**DOI:** 10.1371/journal.pone.0093793

**Published:** 2014-04-15

**Authors:** Andrew F. Taylor, Elizabeth L. Rylott, Christopher W. N. Anderson, Neil C. Bruce

**Affiliations:** 1 Centre for Novel Agricultural Products, Department of Biology, University of York, York, United Kingdom; 2 Institute of Agriculture and Environment, Massey University, Palmerston North, New Zealand; Argonne National Laboratory, United States of America

## Abstract

We have studied the physiological and genetic responses of *Arabidopsis thaliana* L. (Arabidopsis) to gold. The root lengths of Arabidopsis seedlings grown on nutrient agar plates containing 100 mg/L gold were reduced by 75%. Oxidized gold was subsequently found in roots and shoots of these plants, but gold nanoparticles (reduced gold) were only observed in the root tissues. We used a microarray-based study to monitor the expression of candidate genes involved in metal uptake and transport in Arabidopsis upon gold exposure. There was up-regulation of genes involved in plant stress response such as glutathione transferases, cytochromes P450, glucosyl transferases and peroxidases. In parallel, our data show the significant down-regulation of a discreet number of genes encoding proteins involved in the transport of copper, cadmium, iron and nickel ions, along with aquaporins, which bind to gold. We used *Medicago sativa* L. (alfalfa) to study nanoparticle uptake from hydroponic culture using ionic gold as a non-nanoparticle control and concluded that nanoparticles between 5 and 100 nm in diameter are not directly accumulated by plants. Gold nanoparticles were only observed in plants exposed to ionic gold in solution. Together, we believe our results imply that gold is taken up by the plant predominantly as an ionic form, and that plants respond to gold exposure by up-regulating genes for plant stress and down-regulating specific metal transporters to reduce gold uptake.

## Introduction

### Gold Toxicity

Although gold is not required for plant growth, there are soil environments where plants are exposed to this precious metal at significant levels. Gold may exist in soil from natural sources, through the anthropogenic placement of mine tailings in the ecosystem, and more recently from the escalating use of nanoparticles in commercial products [1], [2]. As one of the least reactive chemical elements in the environment, gold exists predominantly in a zero-valent state (Au0). However, studies have demonstrated that gold can be accumulated, to varying degrees, by plant species including *Brassica juncea*, *B. campestris*, *Trifolium repens*, *Sorghum helense*, *Raphanus sativus*, *Kalanchoe serrate* and *Helianthus annuus*, with species-specific differences in the rates of gold uptake and distribution *in planta* [3]. Although gold inhibits plant growth [4]–[8], the exact mechanisms for this inhibition are not well understood. Gold has been reported to covalently bind to cysteine residues and sulfhydryl groups of aquaporins [9], [10]. The inhibition of aquaporins could reduce the water permeability of the root cells, decreasing the ability of the plant to take up water. Gold inhibition of proteins via the disruption of disulfide bonds has also been reported [4], [11] [12] and it is possible that, in addition to disrupting protein structure, gold may displace other essential metals from within proteins inhibiting protein function and leading to metal deficiency symptoms [12].

### Nanoparticle Uptake

Metal nanoparticles are many times more reactive than their bulk equivalents; predominantly a result of the increased surface area to volume ratio for nano sized structures [13]. The emerging industrial use of gold nanoparticles, and their subsequent release into the environment, has led to numerous publications studying the uptake and fate of gold nanoparticles in plants [14]–[16]. Gold nanoparticles between 0.5 nm and 100 nm in diameter have been observed inside tissues of plants exposed to metal nanoparticles [4], [5], [17]–[19]. How metal nanoparticles are taken up by plants is the subject of much conjecture. They could be imported as nanoparticles, or the nanoparticles could be first oxidized to Au(I) or Au(III), dissolved into soil solution, imported as ions and then reduced *in planta* [20]. The uptake of nanoparticles appears to be size selective: aggregates of gold nanoparticles were seen in the root cytoplasm of tobacco exposed to 3.5 nm AuNP spheres, but not when exposure was to 18 nm AuNPs [21]. The literature indicates that, in addition to the debate outlined above, there is much species-dependent variation in the response to nanoparticle treatments [20], [22].

Gold nanoparticles are often coated with compounds such as dextran, polyethylene glycol or bovine serum albumin to improve nanoparticle stability and reduce agglomeration. Whether these coatings affect nanoparticle uptake from soil is not well understood, although it is likely that they are assimilated by soil microorganisms in the rhizosphere and may not affect the process of nanoparticle uptake by plants [19], [23].

### Gold Uptake at the Genetic Level

Several studies have investigated the binding of various states of gold to root membranes [17], [24], but at the genetic level, the mechanism for gold uptake remains unclear. In Arabidopsis, heavy metal ATPases (HMAs), which translocate ions across cell or organelle membranes, have been implicated in the transport of copper and silver and these are elements that share periodicity with gold. For example, HMA5 is involved in copper detoxification in roots (a *hma5* mutant is hypersensitive to copper) [25], while HMA1 and HMA8 transport copper to the chloroplast thylakoids and envelope [26]–[28]. HMA6 transports copper and, to a lesser extent, silver in the inner chloroplast envelope [29].

### Biotechnological Applications for Gold Uptake by Plants

The concept of using plants to recover gold from soil or soil-like media has been proposed as a potentially viable source of the metal for green chemistry [30]. Gold phytoextraction depends on the use of thiocyanate, thiosulfate or cyanide salts to promote gold solubility in soil. While significantly boosting gold accumulation in plants [31], [32], chemically-enhanced gold uptake may not be a long-term, environmentally sustainable mechanism for gold phytoextraction. Improved processes may come from a greater understanding of the biological processes behind gold uptake and toxicity. Biological techniques could be used to select, or engineer, species with an enhanced ability for gold solubilization and uptake. This biological approach, rather than a chemical one, may facilitate the development of phytoextraction as a method to meet demand for gold in a range of industrial, chemical, electronics and medical applications.

To address gaps in our understanding of the response of plants to gold, we have used plant physiology to quantify the toxicity of gold, and gene expression studies to characterize the underlying genetic response of plants to gold. This work was conducted using the model species *Arabidopsis thaliana* L. (Arabidopsis). Alongside this, we used *Medicago sativa* L. (alfalfa) to investigate the uptake response of plants to a range of nanoparticle sizes. The aim of this second phase of our work was to contribute to an understanding of whether gold nanoparticles in plants are the result of direct nanoparticle uptake, or the sequence of oxidation in the root zone, ionic uptake and subsequent *in planta* reduction to nanoparticle forms.

## Materials and Methods

### Chemicals

Gold, as K(AuCl_4_) and AuCl_3_ was obtained from Sigma-Aldrich, St Louis, Missouri, USA. uncoated gold nanoparticles of diameters 7, 18, 48 or 108 nm were obtained from Nanopartz, Colorado, USA. The properties of the nanoparticles are recorded in Table 1.

**Table 1:** Properties of nanoparticles used in this study.

Catalogue number	Listed Nanoparticle Diameter (nm)	Nanoparticles/ml	Concentration (µg/ml)	Molarity (mM)
A11-5-NP-25	7±0.27	1.36×10^13^	47.165	0.239
A11-20-NP-25	18±0.05	7.03×10^11^	41.453	0.210
A11-50-NP-25	49±0.05	3.46×10^10^	41.158	0.209
A11-100-NP-25	108±0.06	3.8×10^9^	48.399	0.246

### Seedling Toxicity Experiments

Surface-sterilized *Arabidopsis thaliana* L. ecotype Col0 (Arabidopsis) seeds were imbibed and stratified for 72 hours in the dark at 4°C then sown onto agar plates containing half-strength Murashige and Skoog (½ MS) [33] medium and 0, 100, 200, 300 and 400 mg/L gold, in the form of K(AuCl_4_) and AuCl_3_. The pH was adjusted to pH 5.7 for all concentrations. Seedlings were grown under 80 µmol m^−2 ^s^−1^ light with a 16 hour photoperiod and 20°C day and 18°C night temperatures.

### Gold Uptake Experiments

For the liquid culture experiments, seven-day-old seedlings were transferred, eight per flasks, to 100 ml conical flasks containing 20 mL of ½ MS medium plus 6.8 g/L sucrose. Plants were grown for 14 days under 20 µmol m^−2 ^s^−1^ light on a rotary shaker at 130 rpm. After this time, the medium was replaced with 20 ml of 6.8 g/L sucrose plus a range of gold concentrations (0, 25, 50, 75, 100, 200, 300 and 400 mg/L), as K(AuCl_4_). Prior to harvest, the plants were rinsed three times with distilled water.

For the sieve-grown experiments, sterilized Arabidopsis seeds were germinated on stainless steel sieves coated with agar containing ½ MS, and suspended on stainless steel legs just above the surface of liquid ½ MS medium enabling the roots to grow into the medium. Plants were grown for six weeks then the medium was replaced with a solution of 200 or 500 mg/L gold, in the form of K(AuCl_4_) for 20 h. Prior to harvest, the roots were rinsed three times with distilled water, then roots and shoots separated before further processing.

### Transmission Electron Microscopy

Samples were fixed in 50 mM phosphate buffer containing 2.5% (v/v) glutaraldehyde and 4% formaldehyde (v/v) for 3.5 hours then washed twice with 100 mM phosphate buffer. A secondary fix of 1% osmium tetroxide in 50 mM phosphate buffer was carried out for 40 minutes then the preparations were washed twice with 50 mM phosphate buffer. Samples were dehydrated then infiltrated and fixed in Spurrs resin (Agar Scientific) (25, 50, 75 and 100%) with overnight polymerization at 70°C. Sections were stained with saturated uranyl acetate and Reynolds lead citrate and viewed using a Tecnai 12 Bio Twin TEM operating at 120 kV. The chemical composition of nanoparticles was confirmed using Energy-dispersive X-ray spectroscopy (EDX) with an Oxford INCA analysis system.

### Determination of Total Gold Concentration

Plant tissues from all gold exposure experiments were dried at 60°C and ashed at 550°C for six hours using a Carbolite AAF 11/7 furnace (Carbolite, Hope, UK). Aqua regia (3∶1 (v/v) 12 M hydrochloric acid : 15.6 M concentrated nitric acid) at a volume of 2.5 mL was added to the ashed samples at 60°C and 7.5 mL of water added. The digest solutions were analyzed by flame atomic absorption spectroscopy (AAS) using a Hitachi Z-5300 Polarized Zeeman Atomic Absorption Spectrophotometer at 242.2 nm. Gold concentrations differed by less than 10% from the reported mean value for a gold-containing plant standard [34] analyzed in parallel with all biomass samples. A gold linear calibration curve over the range 0 to 50 µg/mL was prepared from 1,000 mg/L standard solutions (Sigma) for spectrophotometric determinations. Parallel sample blank solutions were used to check for background gold concentration in solution.

### Statistical Analyses

**Figure 1:**
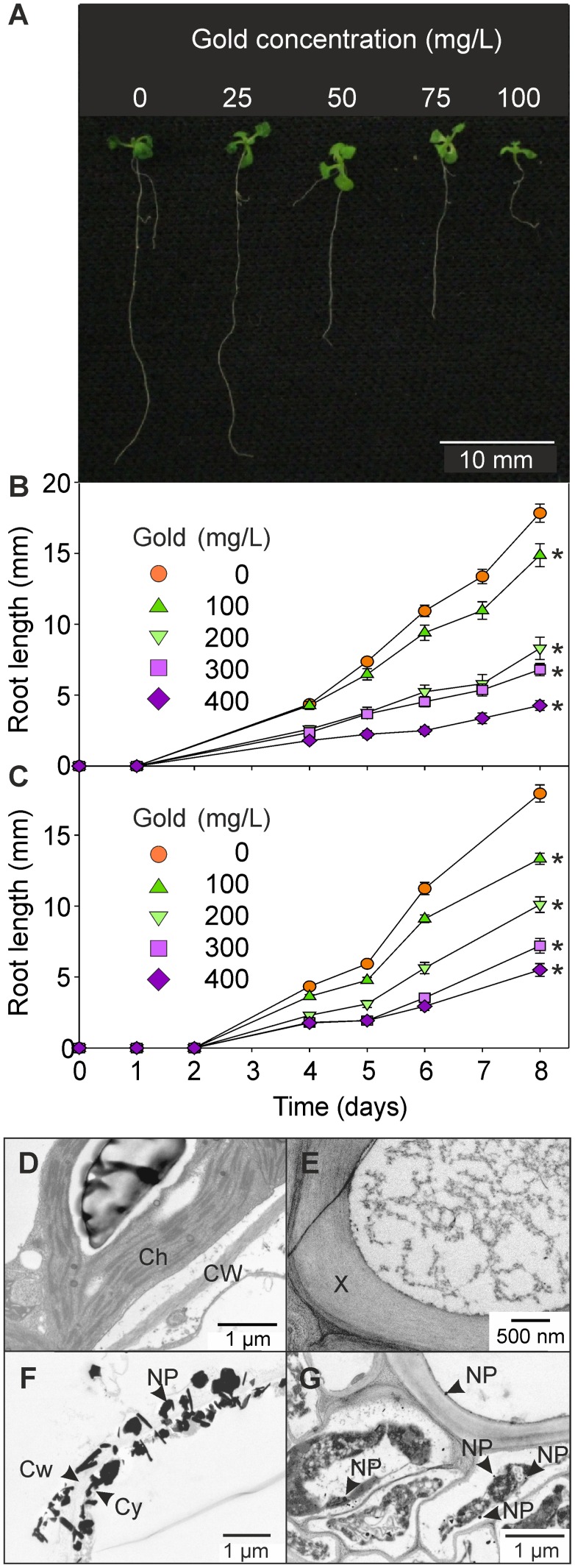
Growth of Arabidopsis seedlings in the presence of gold. (A) Appearance and (B) root length of ten-day-old seedlings grown on agar plates containing a range of gold concentrations, as K(AuCl_4_). (C) Root length of ten-day-old seedlings grown on agar plates containing a range of gold concentrations, as AuCl_3_. Results are the mean from 30 biological replicates ± SD. *  =  significantly different from no gold, at day 8 (*p*<0.001). Transmission electron micrographs showing gold nanoparticles in tissue of plants grown on agar plates containing 100 mg/L gold. (D) Leaf mesophyll and (E) leaf vascular tissue; (F) root cortex and (G) root vascular tissue. NP, nanoparticle; Cy, cytoplasm; Cw, cell wall, X, xylem; Ch, chloroplast.

**Figure 2:**
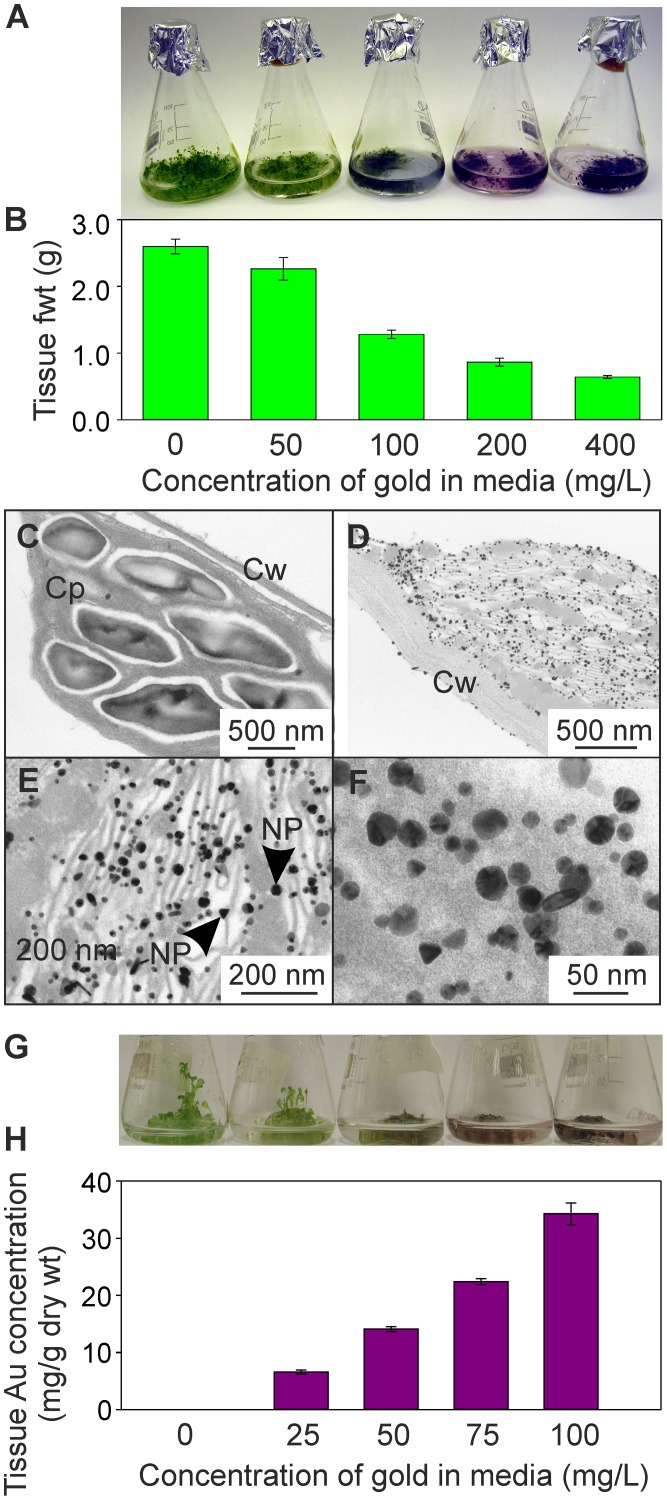
Uptake of gold by Arabidopsis from hydroponic culture. (A) Appearance of three-week-old, liquid-culture grown Arabidopsis plants dosed for 24 h with a range of gold concentrations, as K(AuCl_4_). (B) Levels of gold in plant tissues 24 h after treatment. Results are the mean from five biological replicates ± SE. Transmission electron microscopy images of plants dosed with 200 mg/L gold, as K(AuCl_4_). (C), chloroplasts from untreated plants; (D–F) chloroplasts from NP, nanoparticle; Cy, cytoplasm; Cw, cell wall. (G) Appearance of three-week-old, liquid-culture grown Arabidopsis plants dosed for 24 h with a range of gold concentrations, as K(AuCl_4_). (H) Levels of gold in plant tissues 24 h after treatment. Results are the mean from four biological replicates ± SE.

**Figure 3:**
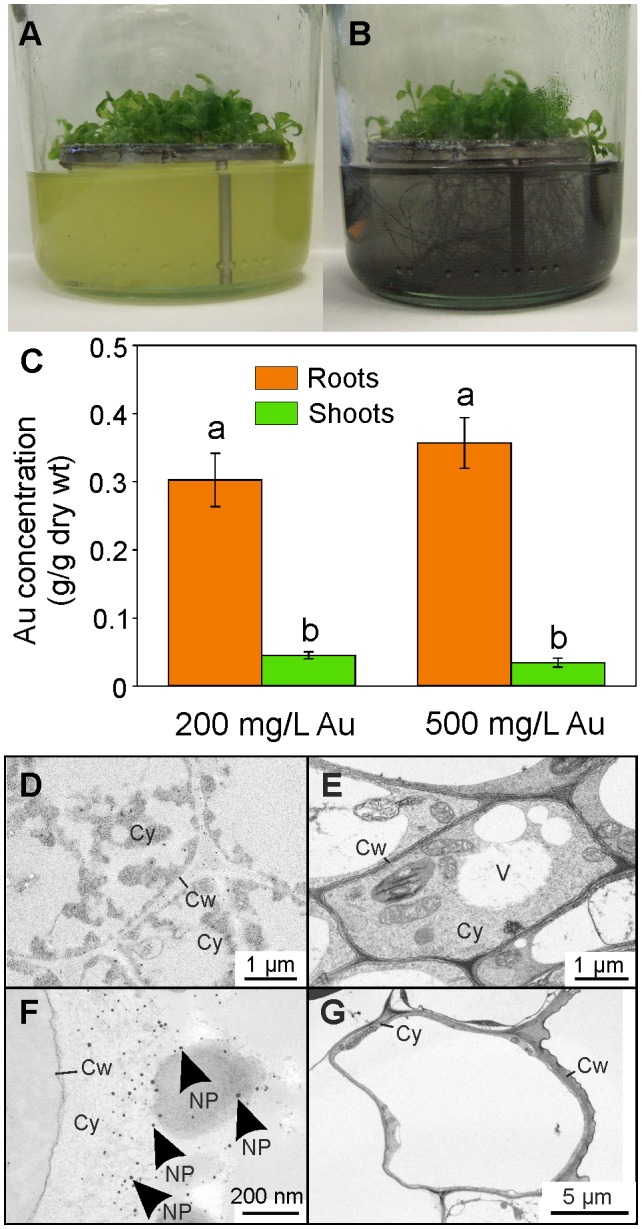
Uptake of gold by Arabidopsis roots. Appearance of six-week-old, hydroponically grown plants (A) before and (B) 24 h after treatment with gold, as K(AuCl_4_). (C) Levels of gold in plant tissues after 24 h, a and b are significantly different from each other (*p*<0.005) within each treatment. Results are the mean from three biological replicates ± SD. Electron micrographs of plant tissues dosed with 500 mg/L gold, as K(AuCl_4_). (D) Leaf mesophyll, (E) leaf vascular tissue, (F) root cortex and (G) root vascular tissue. NP, gold nanoparticle; Cy, cytoplasm; Cw, cell wall, X, xylem; Vw, vascular wall.

Results for the growth of Arabidopsis seedlings in agar plates (Figure 1B and C) are the mean from 30 biological replicates ± SD for each gold concentration. The results measuring uptake of gold by Arabidopsis from hydroponic culture (Figure 2B and 2H) are the mean from five biological replicates ± SE. The results presented for the uptake of gold by Arabidopsis grown on sieves (Figure 3C) are the mean from three biological replicates ± SE. Significant differences for data in Figures 1 and 3 were tested using ANOVA. Analyses were performed using IBM SPSS software (version 20), data were tested for normality using Levene’s test, and variances of groups found to be equal. Statistical analyses of the microarray and qPCR data are presented below.

### Microarray and Quantitative PCR Analysis

**Figure 4:**
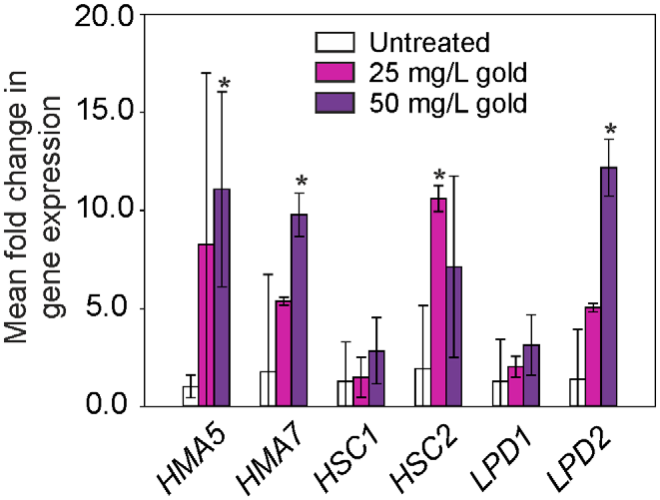
Relative fold induction of gene expression in Arabidopsis root tissue treated with gold. Plants were grown hydroponically for six weeks then the medium was replaced with 25 or 50/L gold, as K(AuCl_4_) for 6 h, and then RNA extracted from roots for qPCR analysis. The data were normalized to the Arabidopsis *ACTIN2* gene and relative fold induction calculated using the ΔΔCt method [36]. Results are the mean from four biological replicates ± SD, * denotes significantly (*p*<0.05) more upregulated than untreated samples.

**Figure 5:**
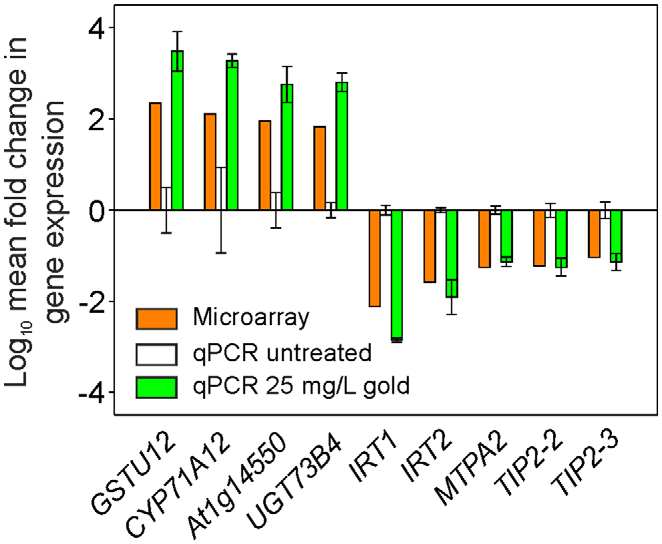
Comparison of qPCR and microarray gene expression levels in Arabidopsis root tissue treated with gold. Plants were grown hydroponically for six weeks then the medium was replaced with 25/L gold, as K(AuCl_4_) for 6 h, and then RNA extracted from roots for qPCR analysis. The data were normalized to the Arabidopsis *ACTIN2* gene and relative fold induction calculated using the ΔΔCt method [36]. Results are the mean from three biological replicates ± SD.

Arabidopsis seeds were germinated and grown hydroponically as described in Kumari *et al.* [35]. Plants were dosed with 25 mg/L gold, in the form of K(AuCl_4_), for 6 h, RNA extracted from roots using a Plant RNeasy kit (Qiagen) and RNA quality verified using an RNA-6000 Nano-Labchip kit and Agilent 2100 bioanalyzer. The cDNA was synthesized and ATH1 chips (Affymetrix, California, USA) hybridized. Data were normalized using Affymetrix GCOS 1.2 software and analyzed using GeneSpring GX10 Expression software (Agilent Technologies, USA). The GeneSpring software, although supplied by Agilent, was designed to analyze data from all of the major platforms including Affymetrix. Differentially expressed genes were identified using a two-class t-test (p<0.05 significance level) with 2-fold cut-off limit. The microarray data were verified using quantitative reverse transcription PCR (qPCR) and the primers listed in Table 2. The qPCR was performed using an ABI 7000 Sequence Detection System (Applied Biosystems) with SYBR green reporter dye. Data were normalized to expression levels of the internal control gene (*actin*) and the comparative ΔΔCt method [36] used to calculate the mean fold change in gene expression of the candidate genes. The results in Figure 4 and 5 are the mean from three and four biological replicates, respectively, ± SD. Significant differences for Figure 5 were tested using ANOVA. Microarray data has been submitted to the Gene Expression Omnibus (GEO) (http://www.ncbi.nlm.nih.gov/geo) accession number GSE46958.

**Table 2:** Primers used for microarray verification qPCR reactions.

Primer Name	Sequence
ATGSTU12F	GATCTTTCCATCCTCCCAACAC
ATGSTU12R	CAACGAAGTGAGCCCAAAAAC
CYP71A12F	TGGTAACCTCCACCAGCTTAGC
CYP71A12R	TGGTCCGTACCGAAGGCTTA
At1g14550F	CTATTC AGGAGCACACACCATAGG
At1g14550R	TGTCGCTTGAGTTCTCGTAAAGC
UGT73B4F	CAACAGAATCCGCGGAGAA
UGT73B4R	GAACAACACAAGGCAAAGGATGA
IRT1F	CTTTGATCACGGTTGGACTTCTAA
IRT1R	AGATCCACGAGTGCCATGTAAA
IRT2F	TCTTTTCAGCCGTTACATTTCG
IRT2R	AGAAGAAAAACATTTGACGATCATGA
MTPA2F	CATAGTTGTAGAAGTCGTTGGAGGAA
MTPA2R	GCAAAGGCTGCAACATCAGA
TIP2;2F	TGACCTTTGCTCTGGTCTACACA
TIP2;2R	TGGTCCCGAGTGAACCTTTC
TIP2;3F	CCCAGCTGGTCTTGTAGCAATT
TIP2;3R	TGTTAGCCGCAATGGAAACTC
ACTINF	TACAGTGTCTGGATCGGTGGTT
ACTINR	CGGCCTTGGAGATCCACAT

### Nanoparticle Uptake Experiments

Alfalfa seeds were germinated and grown aseptically for five days on 1/2½ MS medium. The seedlings were then placed in six-well, polystyrene trays containing 2 ml of gold at a concentration of 50 mg/L in a matrix of sterile water. Gold was either in the form of K(AuCl_4_) (ionic gold) or 7, 18, 48 or 108 nm gold nanoparticles. Sizes of the nanoparticles were as described by the manufacturer (Nanopartz, Colorado, US) (Table 1). Seedlings were treated for 24 hours with shaking at 150 rpm to facilitate nanoparticle suspension; no sediments were visible. After 24 hours of treatment, roots were fixed, sectioned and analyzed by TEM as described above.

## Results and Discussion

### Toxicity of Gold to Arabidopsis

A range of gold concentrations was tested with the aim of establishing the concentration at which Arabidopsis exhibits gold-toxicity symptoms. While germination was unaffected at any of the K(AuCl_4_) concentrations tested (data not shown), subsequent root growth was inhibited with increasing K(AuCl_4_) concentration (Figure 1A). Subsequent ANOVA tests revealed that after eight days of growth there was a significant effect of K(AuCl_4_) or AuCl_3_ on seedling root length at all concentrations tested (Figure 1B and C). At 100 mg/L gold, the highest concentration of K(AuCl_4_) or AuCl_3_ tested, seedling root length was reduced by approximately 75%. To see if there were concentrations of other metals (such as cadmium, lead and nickel) that could account for, or contribute to the observed toxicity symptoms, the K(AuCl_4_) was analyzed using inductively coupled plasma optical emission spectroscopy (ICP OES) (Medac Ltd, Surrey). The results showed that the K(AuCl_4_) comprised 50.33% Au and 6.93% K. Contaminating metals were considered to be below toxicity threshold levels in the experiments presented here. In a 100 mg/L gold as K(AuCl_4_) solution, potential contaminant concentrations (ppb) were: Ag, 0.4; Al, 0.8; B,0.4; Ca, 6.9; Co, 9.4; Cu, 0.6; Fe, 0.3; Hg, 0.2; Mg, 0.4; Na, 4.5; Ni, 0.6; P, 0.3; Pt, 2.1; Ru, 0.1; Sb, 1.4; Se, 1.3; Si, 0.2; W, 0.4; Zn, 7.2 (all other metals <0.1 ppb).

As ½ MS salts were added to the media in these experiments, it is unlikely that the toxicity is due to the potassium or chloride ions (½ MS includes 950 mg/L KH_2_PO_4_, 85 mg/L KH_2_PO_4_ and 220 mg/L CaCl_2_.2H_2_O), thus we conclude that the toxicity is directly due to gold. Transmission electron micrographs (TEMs) of the seedlings revealed the root cortex and vascular tissues to be densely packed with electron-dense nanoparticles which were absent from leaf tissues (Figure 1D–G). This was subsequently identified as gold using EDX.

### Gold Uptake from Liquid Medium by Arabidopsis

To investigate gold uptake by Arabidopsis, plants were grown in liquid culture. After 3 weeks of growth, the plants had produced predominantly shoot and leaf tissues, with approximately 5% dry weight comprising root tissues. After this time, the medium was replaced with water containing a range of gold concentrations (0, 50, 100, 200 and 400 mg/L), in the form of K(AuCl_4_)). The concentrations chosen encompassed the range at which toxicity symptoms were observed in the seedlings grown on plates in Figure 1B. After 24 hours, a purple coloration, indicative of the presence of gold nanoparticles [37], was observed on the root and shoots of the plants, the coloration deepening with increasing gold media concentration (Figure 2A). The fresh weight biomass of Arabidopsis decreased with exposure to increasing concentrations of K(AuCl_4_) (Figure 2B). At the highest concentration of gold treatment (400 mg/L gold), TEM analysis revealed that nanoparticles were present in both the shoot chloroplasts and cytoplasm, and root tissues (Figure 2C–E). A range of differently shaped particles of diameters between five and 30 nm was observed (Figure 2F). A second liquid culture experiment (Figure 2G and H) was conducted to measure the levels of gold accumulated in plants dosed with slightly lower concentrations (0, 25, 50, 75 and 100 mg/L) of gold, in the form of K(AuCl_4_), for 24 hours. This experiment showed that the plants accumulated increasing concentrations of gold in the plant tissues, up to 34.3±1.9 mg gold per g dry weight. This indicates that even at the 100 mg/L gold treatment, the uptake capacity was not saturated; however, although the tissues were rinsed with distilled water prior to harvest, some of the total gold measured will have been adsorbed to the outside of the plant tissues.

To establish whether gold is translocated from the roots to the aerial parts, Arabidopsis plants were grown hydroponically using a sieve to separate leaf biomass from the medium, as shown in Figure 3A. The hydroponic medium was replaced with a solution of 200 or 500 mg/L gold, in the form of K(AuCl_4_). After 20 hours, the roots and media were a purple color (Figure 3B) and gold was detected in the shoot tissues confirming uptake and translocation of the element to the aerial biomass. For both media gold concentrations there was a significantly (p<0.005) higher concentration of gold in the roots relative to the shoots. This concentration was by a factor of 6.7 for the 200 mg/L treatment and 10.1 for the 500 mg/L treatment although no quantification was made of the proportion of gold that might be adsorbed to the root surface. At both media concentrations, the gold content of the roots comprised 30% of the total dry weight (Figure 3C). As levels of gold in the shoots were not significantly different between 200 and 500 mg/L, we assume that gold uptake capacity was saturated at 200 mg/L within the 20 hours of the experiment. Subsequent TEM analysis demonstrated that gold nanoparticles, confirmed using EDX, were present in the root cytoplasm, but were absent from the leaf tissues (Figure 3D–G).

Our data show that Arabidopsis can accumulate ionic gold from solution through roots and translocate the metal to aerial biomass. However, the absence of nanoparticles in the shoots was unexpected: When plants were grown in liquid culture and exposed to a gold concentration of 100 mg/L gold, the tissue gold concentration (which is predominantly shoot tissues) was 34.3 mg gold/g dry weight and nanoparticles were observed (Figure 2D–F). When the plants were grown on sieves and exposed to 200 and 500 mg gold/L of solution, although the gold content in the shoot tissues was about 8 to 10-fold higher than in the liquid culture experiment, nanoparticles were not detected (tissue concentrations of 302.4±39.0 and 356.7±37.0 mg gold/g shoot dry weight for the 200 and 500 mg/L treatments respectively). This suggests that gold entering the submerged shoot tissues directly through the shoot epidermis will form nanoparticles, whereas gold translocated from the root is maintained in an oxidized form, or possibly as nanoparticles below the resolution of TEM.

### Arabidopsis Gene Expression in Response to Gold

To identify candidate transporter genes that are potentially involved in the uptake of gold, we conducted a microarray study. There are no reported microarray expression studies for gold treatment of plants, although microarray research in the bacterium *Cupriavidus metallidurans* identified genes up-regulated in response to gold treatment [38]. Among the most highly up-regulated, were genes encoding products implicated in metal homeostasis. The closest orthologs in Arabidopsis, *HMA5* (At1g63440), *mtLPD1* (At1g48030) and *mtHSC1* (At4g37910) were found to be involved in the response, or uptake, of a range of transition metals [25], [39], [40]. The expression of these Arabidopsis orthologs, along with related Arabidopsis genes; *HMA7* (At5g44790), *mtLPD2* (At3g17240) and *mtHSC2* (At5g09590) were used as calibrators for the microarray experiment. Hydroponically grown plants were exposed to a gold concentration of 25 mg/L or 50 mg/L, in the form of K(AuCl_4_), for six hours, then qPCR was conducted using cDNA derived from root tissue. Gene expression values were all higher in the gold treated samples than the controls, although only four of the six genes tested showed significantly increased levels (*p*<0.05) (Figure 4). Microarray studies were subsequently conducted using the 25 mg/L gold treatment. Analysis of the results revealed that 851 genes were down-regulated and 869 up-regulated more than two-fold in response to the gold treatment. The 25 most down-regulated and 25 most up-regulated genes are listed in Tables 3 and 4 respectively. The reliability of the microarray data was validated by qPCR analysis which revealed a similar expression pattern to the microarray data for four up-regulated and five down-regulated genes (Figure 5). The toxicity of gold under these conditions is quantified in the up-regulation of a large number of genes classically upregulated in response to abiotic stresses such as glutathione transferases, cytochromes P450, glucosyl transferases and peroxidases [41]–[43]. Maser et al. [44] reported that over 5% of the Arabidopsis genome encodes membrane transport proteins, including over 150 cation transport proteins. A striking feature of the dataset was the large percentage of down-regulation of genes involved in metal transport, with divalent cation transporters highly down-regulated (Table 5). Iron Regulated Transporter *IRT1* was the most down regulated gene in the microarray (132-fold) and *IRT2* expression was also decreased (38.4-fold). The nickel transporter *ATIREG2* and the copper transporter, *COPT2* were also down-regulated 23.6 and 22.8-fold respectively. Both essential and non-essential metal ions can become phytotoxic when present in excess. To prevent this, plants have evolved mechanisms to control uptake. That gold is able to trigger these control mechanisms, down-regulating divalent cation transporters to reduce gold uptake and subsequent toxicity, implies that some proportion of gold in the environment exists in a cation form, and that these transporters have activity towards gold. While plant transporters that take up gold have not been found, the ITR transporters identified here are able to take up a wide range of metal cations; cadmium, cobalt, manganese, zinc and copper [45]–[48]. This broad substrate range could extend to gold. In contrast, the HMA and COPT transporters, which also response to gold treatment, have a narrow substrate range, importing copper and silver; metals closely related to gold. Yeast expression studies could be used to investigate the ability of these transporters to take up gold.

**Table 3:** Twenty-five genes most down-regulated in response to gold treatment.

Fold change	Locus	Gene name	Description
132.4	At4g19690	IRT1	Fe(II) transport protein
106.7	At5g46900		protease inhibitor/seed storage/lipid transfer protein
67.6	At5g04950		nicotianamide synthase
55.8	At1g08090	ATNRT2∶1	high-affinity nitrate transporter
55.6	At4g12550	AIR1	putative cell wall-plasma membrane disconnecting CLCT protein (AIR1A)
43.2	At1g73120		hypothetical protein
38.4	At4g19680	IRT2	Fe(II) transport protein
37.2	At3g12900	MJM20	oxidoreductase, 2OG-Fe(II) oxygenase family protein
32.8	At3g12820	AtMYB10	myb-related protein
32.6	At1g49860	ATGSTF14	glutathione S-transferase
30.8	At4g31940	CYP82C4	cytochrome P450
26.4	At3g61930		hypothetical protein with unknown function
25.8	At5g04730		hypothetical protein with unknown function
25.6	At3g19430		protein with unknown function; abundant in late embryogenesis
23.6	At3g46900	COPT2	copper transport protein
23.4	At3g45710	T6D9.40	proton-dependent oligopeptide transport (POT) family protein
23.3	At5g54370		protein with unknown function; abundant in late embryogenesis
23.2	At2g01530	MLP329	unknown protein related to major latex proteins, involved in copper binding
22.8	At5g03570	FPN2	tonoplast localized nickel transport protein
21.6	At4g22460		putative protease inhibitor/seed storage/lipid transfer protein
21.3	At3g44990	XTR8	xyloglucan endo-transglycosylase
19.9	At2g28160	FIT1	putative bHLH transcription factor regulating iron uptake responses
19.2	At3g18450		hypothetical protein with unknown function
19.2	At3g50740	UGT72E1	UTP-glucose glucosyltransferase
19.0	At1g34760	GRF11	encodes a 14-3-3 protein. Binds H+ -ATPase in response to blue light

**Table 4:** Twenty-five genes most up-regulated in response to gold treatment.

Fold change	Locus	Gene name	Description
291.1	At3g16530		putative lectin
233.9	At1g26380		hypothetical protein containing FAD-binding domain
220.2	At1g69920	ATGSTU12	glutathione transferase
133.9	At5g22300	NIT4	nitrilase specific for beta-cyano-L-alanine
132.2	At5g40990	GLIP1	GDSL-motif lipase
127.2	At2g30750	CYP71A12	cytochrome P450
122.0	At2g43000	ANAC042	transcription factor with NAC domain
102.8	At1g64160		dirigent protein
93.4	At4g31970	CYP82C2	cytochrome P450
88.7	At1g14550		anionic peroxidase
87.6	At1g66690		S-adenosyl-L-methionine: carboxyl methyltransferase family protein
86.9	At2g35980	YLS9	similar to harpin-induced protein hin1
85.2	At3g46230	ATHSP17.4	small heat shock protein
82.3	At1g69930	ATGSTU11	glutathione transferase
78.1	At3g60120	BGLU27	beta-glucosidase
76.1	At4g37290		hypothetical protein with unknown function
74.8	At5g39580		putative peroxidase
74.1	At3g26200	CYP71B22	cytochrome P450
74.1	At5g12030	ATHSP17.6	small heat shock protein
69.5	At2g26560	PLP2	lipid acyl hydrolase
67.7	At2g28210	ATACA2	alpha carbonic anhydrase
66.6	At2g15490	UGT73B4	glucosyltransferase
63.5	At1g05680	UGT74E2	glucosyltransferase
62.3	At1g53540	ATHSP17.6C	small heat shock protein
55.4	At3g54150		putative methyltransferase

**Table 5:** Changes in regulation of divalent cation transporters in response to gold.

Fold change	Regulation	Locus	Gene	Description
132.3	Down	At4g19690	IRT1	Cadmium, copper, iron, manganese and zinc transporter
38.4	Down	At4g19680	IRT2	Iron and zinc transporter
23.6	Down	At3g46900	COPT2	Copper transmembrane transporter
22.8	Down	At5g03570	ATIREG2	Nickel transmembrane transporter
17.9	Down	At3g58810	MTPA2	Zinc ion transmembrane transporter
13.7	Down	At3g58060	MTPc3	Cation efflux family protein
4.4	Down	At4g30120	HMA3	Heavy metal ATPase 3
3.2	Down	At1g64170	ATCHX16	Sodium: hydrogen antiporter
3.1	Down	At5g26820	ATIREG3	Iron regulated protein 3
3.0	Down	At1g80830	NRAMP1	Manganese transmembrane transporter
4.6	Up	At3g51860	CAX3	Calcium cation antiporter

Expression of FIT1, a transcription factor involved in the regulation of iron, is significantly down-regulated (20-fold) in our microarray study. Expression of FIT1 is induced by iron deficiency which suggests that expression would be low when iron is unlimited. Twelve of the 25 most down-regulated genes in our microarray are thought to be under the regulation of FIT1 and eight of the ten most down-regulated divalent cation transporters presented in Table 5 (*IRT1*, *IRT2*, *COPT2*, *ATIREG2*, *MTPA*, *MTPc3*, *HMA3* and *NRAMP*) are likely to be under the regulation of FIT1 [49].

Eleven aquaporin genes were also down-regulated by the gold treatment (Table 6). Aquaporins are integral membrane pore proteins that selectively conduct water molecules in and out of the cell. The inhibition of water uptake, and movement throughout the plant, could explain the toxicity symptoms we observed in gold-treated Arabidopsis seedlings (Figure 1A). Gold has previously been shown to inhibit aquaporin function [50], and studies have shown that silver, an element with chemical similarity to gold, and mercury inhibit aquaporins via the covalent modification of cysteine residues [50], [51].

**Table 6:** Changes in regulation of aquaporins in response to gold.

Fold Change	Regulation	Locus	Gene
13.91	Down	At1g31885	NIP3;1
3.55	Down	At3g61430	PIP1;1
2.84	Down	At1g01620	PIP1;3
9.69	Down	At4g23400	PIP1;5
8.03	Down	At5g60660	PIP2;4
2.04	Down	At4g35100	PIP2;7
7.12	Up	At5g18290	SIP1;2
5.39	Down	At2g36830	TIP1;1
5.79	Down	At3g26520	TIP1;2
8.94	Down	At3g16240	TIP2;1
16.84	Down	At4g17340	TIP2;2
10.79	Down	At5g47450	TIP2;3

### Gold Uptake and Nanoparticle Formation in Alfalfa

**Figure 6:**
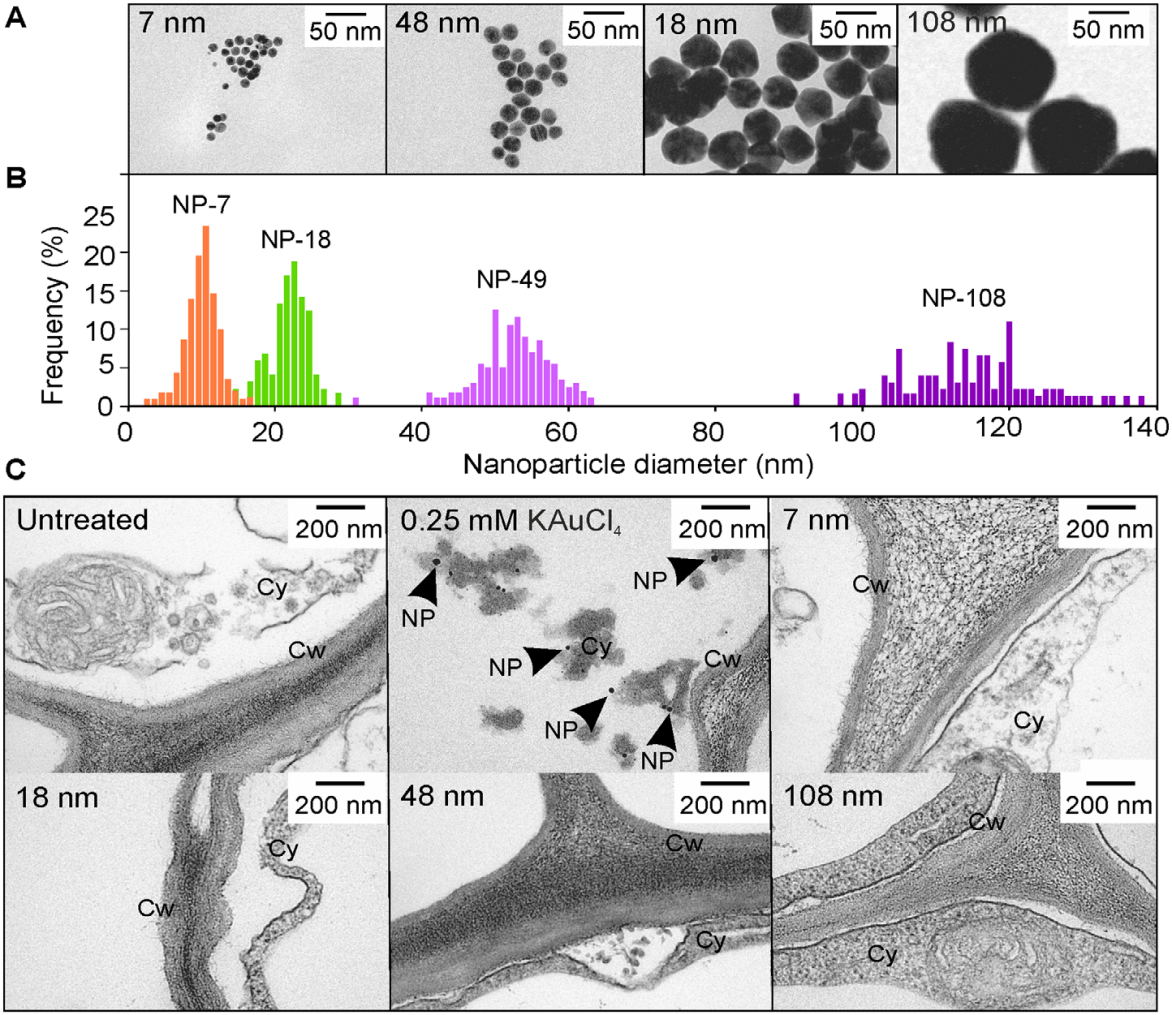
Nanoparticle uptake by alfalfa seedlings. (A) Transmission electron microscopy and (B) size frequency distribution of commercially obtained gold nanoparticles. (C) Transmission electron microscopy of five-day-old alfalfa seedlings 24 h after treatment with ionic gold, as K(AuCl_4_), or nanoparticles. Nanoparticle treatments were of equivalent molarity to the control 50 mg/L gold, as K(AuCl_4_). Three seedlings were sectioned at each treatment. NP, nanoparticle; Cy, cytoplasm; Cw, cell wall.

There is considerable debate as to the mode of uptake of metal nanoparticles [20]; are the nanoparticles taken up directly, or as ions which are then assembled *in planta*? To contribute to this debate, we hypothesized that if the nanoparticles are taken up directly, the diameter frequency distribution will match that of the original nanoparticles. Due to the relatively small size of Arabidopsis roots, we conducted an experiment to test this hypothesis using five-day-old alfalfa seedlings (not Arabidopsis), as similar results for nanoparticle formation in Arabidopsis have been observed in alfalfa [17], [18]. The alfalfa plants were treated with uncoated nanoparticles ranging from 7 to 100 nm in diameter (Figure 6A and B). Plants were exposed to gold nanoparticles at a molar concentration equivalent to a gold concentration of 50 mg/L, with K(AuCl_4_) at a concentration of 50 mg/L used for the ionic control treatment. After 24 hours, biomass samples were removed and prepared for TEM analysis. Nanoparticles were only found in roots exposed to the ionic gold control. No nanoparticles were found in the roots of the plants exposed to nanoparticles ranging from 7 to 100 nm in diameter (Figure 6C to H). The experiment was repeated using the same number of nanoparticles of 7, 18, 48 or 108 nm diameter (3.65×10^8^ nanoparticles per mL; concentration of nanoparticles was the variable). Again, nanoparticles were not found in the tissues of plants exposed to nanoparticles, whereas nanoparticles were found in the ionic controls.

Nanoparticles appear to form in plant tissues after the uptake and translocation of gold ions [4], [7], [52], [53]. However, the mechanism for nanoparticle formation within plants is unknown. Studies of silver nanoparticle formation in plants found that ions are taken up though the roots and then reduced to nanoparticles within the plant [54]. Once formed, it appears that the nanoparticles can move via the transpiration stream through the plant to the aerial tissues [55], [56] and Wang et al. (2012) reported xylem- and phloem-based transport of copper oxide nanoparticles [57].

Our results indicate that, under the conditions we used, nanoparticles between 5–100 nm in diameter are not directly taken up by alfalfa plants as gold nanoparticles were only observed in plants exposed to ionic gold in solution. It is possible that nanoparticles less than 5 nm diameter would be taken up; a study has shown uptake of 3.5 nm gold nanoparticles by tobacco [21]. The absence of TEM-identified nanoparticles in the roots and shoots of alfalfa plants exposed to gold nanoparticles also indicates that gold nanoparticles between 5–100 nm in the growth media are not being solubilized, transported as ions, and then reduced into nanoparticles *in planta*. However, the validity of these conclusions is limited to the experimental conditions used in the study. Chemical reactivity is known to increase with decreasing nanoparticle diameter [37] and Anderson (2013) proposed that smaller (<5 nm) particles might be readily oxidized to gold ions under the geochemical conditions of the rhizosphere for uptake and subsequent reduction within plant tissues [58]. This could be tested by measuring the diameter of <5 nm particles in both the growth medium and plants tissues before and after uptake.

## Conclusion

Our study on the physiological and genetic responses of plants to gold has revealed that gold in solution is taken up by Arabidopsis with approximately 10 to 15% translocated to the shoot tissues. Our studies have shown that gold entering the shoot tissues directly, via passive uptake, can accumulate as nanoparticles in the shoot. We also show that gold taken up by the root can form nanoparticles in the root cells. However, gold translocated to the aerial tissues appears to be restricted from forming nanoparticles in these cells. Our studies using alfalfa indicate that 5 to 100 nm diameter nanoparticles are not directly taken up by plant cells. We believe that the sequence of gold oxidation in the rhizosphere, uptake of the ionic gold, and the reduction of gold *in planta* to create nanoparticles is a plausible route for gold nanoparticle formation.

Our gene expression studies show that gold affects the transcription of a discreet set of genes, particularly cation transporters. Many of these transporters are already known to be involved in the uptake of metals, particularly heavy metals. This indirect evidence further suggests that gold is taken up by the plant as an ionic form, via specific metal transporters. The high number of ion transporters responding to gold, in tandem with the broad range in cation substrate specificity means that there could be a high degree of functional overlap in transporters with activity towards gold.
